# Molecular basis of the human ribosomopathy Shwachman-Diamond syndrome

**DOI:** 10.1016/j.jbior.2017.09.002

**Published:** 2018-01

**Authors:** Alan J. Warren

**Affiliations:** a Cambridge Institute for Medical Research, Cambridge, UK; b The Department of Haematology, University of Cambridge, Cambridge, UK; c Wellcome Trust-Medical Research Council Stem Cell Institute, University of Cambridge, Cambridge, UK

**Keywords:** SBDS, DNAJC21, eIF6, Shwachman-Diamond syndrome, Ribosome, Myelodysplastic syndromes, rRNA, ribosomal RNA, rDNA, ribosomal DNA, ITS1, internal transcribed spacer 1, ITS2, internal transcribed spacer 2

## Abstract

Mutations that target the ubiquitous process of ribosome assembly paradoxically cause diverse tissue-specific disorders (ribosomopathies) that are often associated with an increased risk of cancer. Ribosomes are the essential macromolecular machines that read the genetic code in all cells in all kingdoms of life. Following pre-assembly in the nucleus, precursors of the large 60S and small 40S ribosomal subunits are exported to the cytoplasm where the final steps in maturation are completed. Here, I review the recent insights into the conserved mechanisms of ribosome assembly that have come from functional characterisation of the genes mutated in human ribosomopathies. In particular, recent advances in cryo-electron microscopy, coupled with genetic, biochemical and prior structural data, have revealed that the SBDS protein that is deficient in the inherited leukaemia predisposition disorder Shwachman-Diamond syndrome couples the final step in cytoplasmic 60S ribosomal subunit maturation to a quality control assessment of the structural and functional integrity of the nascent particle. Thus, study of this fascinating disorder is providing remarkable insights into how the large ribosomal subunit is functionally activated in the cytoplasm to enter the actively translating pool of ribosomes.

## 1 Introduction

Shwachman-Diamond syndrome (SDS, OMIM #260400) is a fascinating autosomal recessive disorder associated with bone marrow failure and an increased risk of transformation to myelodysplastic syndrome (MDS) and acute myeloid leukaemia (AML). SDS is also characterised by multiple developmental anomalies including poor growth, exocrine pancreatic insufficiency, skeletal abnormalities (including metaphyseal chondrodysplasia, rib cage dysplasia and osteopenia) and cognitive impairment. However, registry data indicate that the phenotypic spectrum of this rare disease (estimated prevalence of 1 in 77,000 births) is broad (Myers et al., 2014). SDS is most commonly associated with biallelic mutations in the eponymous *SBDS* gene (Boocock et al., 2003), named after the US physician Harry Shwachman, the British ophthalmologist Martin Bodian and the American paediatrician Louis Diamond who reported the syndrome in 1964 (Shwachman et al., 1964). However, Nezelof and Watchi first described SDS as “congenital lipomatosis of the pancreas” in 1961 in two children with exocrine pancreatic insufficiency and leucopenia (Nezelof and Watchi, 1961).

In a French cohort of 102 SDS patients, the cumulative incidence of MDS/AML was 18.8% at 20 years and 36.1% at 30 years of age (Donadieu et al., 2012). Among young adults (18–40 years old) transplanted for MDS who were enrolled in the Center for International Blood and Marrow Transplant Research (CIBMTR) repository between 2005 and 14, 4% were discovered to have germline compound heterozygous mutations in the *SBDS* gene (Lindsley et al., 2017) with concurrent somatic biallelic loss-of-function *TP53* variants. These SDS patients, who were mostly undiagnosed prior to transplant, had a remarkably poor prognosis (median survival of 1.2 years). Thus, loss-of-function *TP53* mutations appear to be biologically important for clonal progression and transformation to haematological malignancy in SDS. Although the incidence is unknown, a limited number of case reports have highlighted a range of solid tumours presenting at a young age in individuals with SDS (Dhanraj et al., 2013, Nakaya et al., 2014, Sack et al., 2011, Sharma et al., 2014, Singh et al., 2012, Verbrugge and Tulchinsky, 2012).

Since the recognition of SDS as a distinct clinical entity in 1961, there have been several key advances in the characterisation of the disease. A major milestone was the identification of biallelic mutations in the *SBDS* gene in 90% of individuals with SDS (Boocock et al., 2003). A second milestone was the discovery that SBDS functions as a cofactor for elongation factor-like GTPase 1 (EFL1) in removing the anti-association factor eIF6 from the subunit joining face of the large (60S) ribosomal subunit in the final step of late cytoplasmic maturation (Finch et al., 2011, Menne et al., 2007, Wong et al., 2011). A third major step was visualising the human SBDS and EFL1 proteins bound to 60S ribosomal subunits carrying endogenous eIF6 using single-particle cryo-electron microscopy (cryo-EM) (Weis et al., 2015). The structures suggest that eIF6 is removed by a cofactor-dependent conformational switching mechanism and allow SDS-associated disease mutations to be interpreted for the first time in a ribosomal context. Finally, the discovery of mutations in the 60S ribosome assembly factor *DNAJC21* (yeast *JJJ1*) in SDS (Dhanraj et al., 2017, Tummala et al., 2016) reveals genetic heterogeneity in this disorder, but supports the original hypothesis that the primary defect in SDS is impaired maturation of the large ribosomal subunit (Menne et al., 2007).

The aim of this review is to discuss the considerable expansion in our understanding of the molecular basis of SDS and to illustrate how elucidation of the function of the genes mutated in this disorder is providing important new mechanistic insights into the fundamental conserved process of ribosome assembly and its quality control. A key remaining challenge is to explain how mutations that affect protein synthesis in all cells cause such tissue-specific abnormalities. Finally, we need to harness new mechanistic insights to develop novel therapeutics that will improve the outcomes for SDS patients and their families.

## 2 Genetic heterogeneity in Shwachman-Diamond syndrome

In 2003, a positional cloning strategy identified biallelic mutations in the highly conserved *SBDS* gene in the majority of individuals with SDS (Boocock et al., 2003). Pathogenic mutations arise as a consequence of gene conversion due to recombination between *SBDS* and an unprocessed pseudogene located in a distal paralogous duplicon. More than 90% of affected individuals carry one of three common pathogenic SBDS variants on one allele in exon 2: 183_184TA > CT, 258+2T > C, or the combination of 183_184TA > CT + 258 +2T > C. The mutation 258+2T > C disrupts the donor splice site of intron 2, while the dinucleotide alteration, 183–184TA→CT, introduces an in-frame stop codon (K62X). Although most parents of children with SDS are carriers, about 10% of *SBDS* mutations arise de novo (Steele et al., 2014). In addition, more than 40 novel sequence variants have been described in individuals who are compound heterozygotes with one of the three common gene conversion alleles (Boocock et al., 2003, Donadieu et al., 2012, Erdos et al., 2006, Finch et al., 2011, Kuijpers et al., 2005, Makitie et al., 2004, Nicolis et al., 2005, Shammas et al., 2005).

No individuals have been identified who are homozygous for the dinucleotide variant (183–184TA→CT) that introduces an in-frame stop codon, suggesting that complete absence of functional SBDS protein is incompatible with life (Boocock et al., 2003). Consistent with this hypothesis, the *SBDS* gene is essential at the cellular level in *Dictyostelium discoideum* (Wong et al., 2011) and is required for viability in zebrafish (Provost et al., 2012) and mice (Finch et al., 2011, Zhang et al., 2006). Thus, the SDS disease phenotype is associated with the expression of at least one hypomorphic *SBDS* allele. Full-length SBDS protein is still detectable, albeit at low levels, in cells from individuals with SDS (Austin et al., 2005, Wong et al., 2011).

As 5–10% of patients clinically diagnosed with SDS are negative for *SBDS* mutations, it was suspected that one or more additional genes might be linked to this disorder. The molecular pathology of SDS has indeed now been expanded to include biallelic mutations in *DNAC21* (Dhanraj et al., 2017, Tummala et al., 2016), the human homologue of the yeast Jjj1 protein that functions in cytoplasmic maturation of the 60S ribosomal subunit (Meyer et al., 2007, Meyer et al., 2010). Biallelic *DNAJC21* mutations were identified in four individuals with global bone marrow failure, intrauterine growth retardation and/or short stature, one of whom developed AML (megakaryocytic subtype, AML-M7) at 12 years of age (Tummala et al., 2016). In a second study, four individuals from three unrelated families who had been diagnosed clinically with SDS were found to carry biallelic *DNAJC21* mutations (Dhanraj et al., 2017). Interestingly, the two studies suggest an association between biallelic *DNAJC21* mutations and retinal dystrophy. In summary, SDS is a genetically heterogeneous disorder caused by mutations that target a common pathway involved in maturation of the large ribosomal subunit. It is likely that additional gene variants may be identified in SDS in the near future by exome sequencing.

## 3 Overview of eukaryotic ribosome assembly

The process of ribosome assembly is summarised to provide a biological framework for understanding the molecular basis of SDS. The synthesis of new ribosomes is a fundamental conserved process involving the coordinated expression and assembly of 80 ribosomal proteins, 4 ribosomal RNAs and 76 small nucleolar RNA molecules by a cast of around 200 assembly factors. The process of ribosome biogenesis is initiated in the nucleolus with transcription of the pre-ribosomal RNA and formation of the 90S particle (also known as the small subunit (SSU) processome) (Chaker-Margot et al., 2017, Dragon et al., 2002, Grandi et al., 2002, Kornprobst et al., 2016, Sun et al., 2017). Endonucleolytic cleavage of the pre-rRNA generates the earliest pre-40S and pre-60S subunits (Goldfarb and Cech, 2017, Trapman et al., 1975) that are subsequently exported independently across the nuclear pore. Release of the placeholder GTPase Nog2 is coupled to recruitment of the essential nuclear export adaptor Nmd3, providing a checkpoint for ribosome export (Matsuo et al., 2013). Nmd3 carries a C-terminal leucine-rich nuclear export sequence that recruits the RanGTP-dependent export receptor Crm1 to the pre-60S subunit (Gadal et al., 2001, Ho et al., 2000). Additional Crm1-independent adaptor proteins, including Arx1, Rrp12, Ecm1, Bud20 and the Mex67-Mtr2 heterodimer form part of the export machinery for the pre-60S subunit. Arx1 transiently interacts with FG-repeat nucleoporins but despite structural homology to methionine aminopeptidases, Arx1 lacks enzyme activity (Bradatsch et al., 2007, Kowalinski et al., 2007). The heterodimeric Mex67-Mtr2 export receptor binds pre-60S ribosomes in the neighbourhood of the nascent P stalk and within the 3’ region of the 5.8S rRNA (Sarkar et al., 2016). However, the precise roles of the other assembly factors implicated in 60S export remain unclear.

**Fig. 1:**
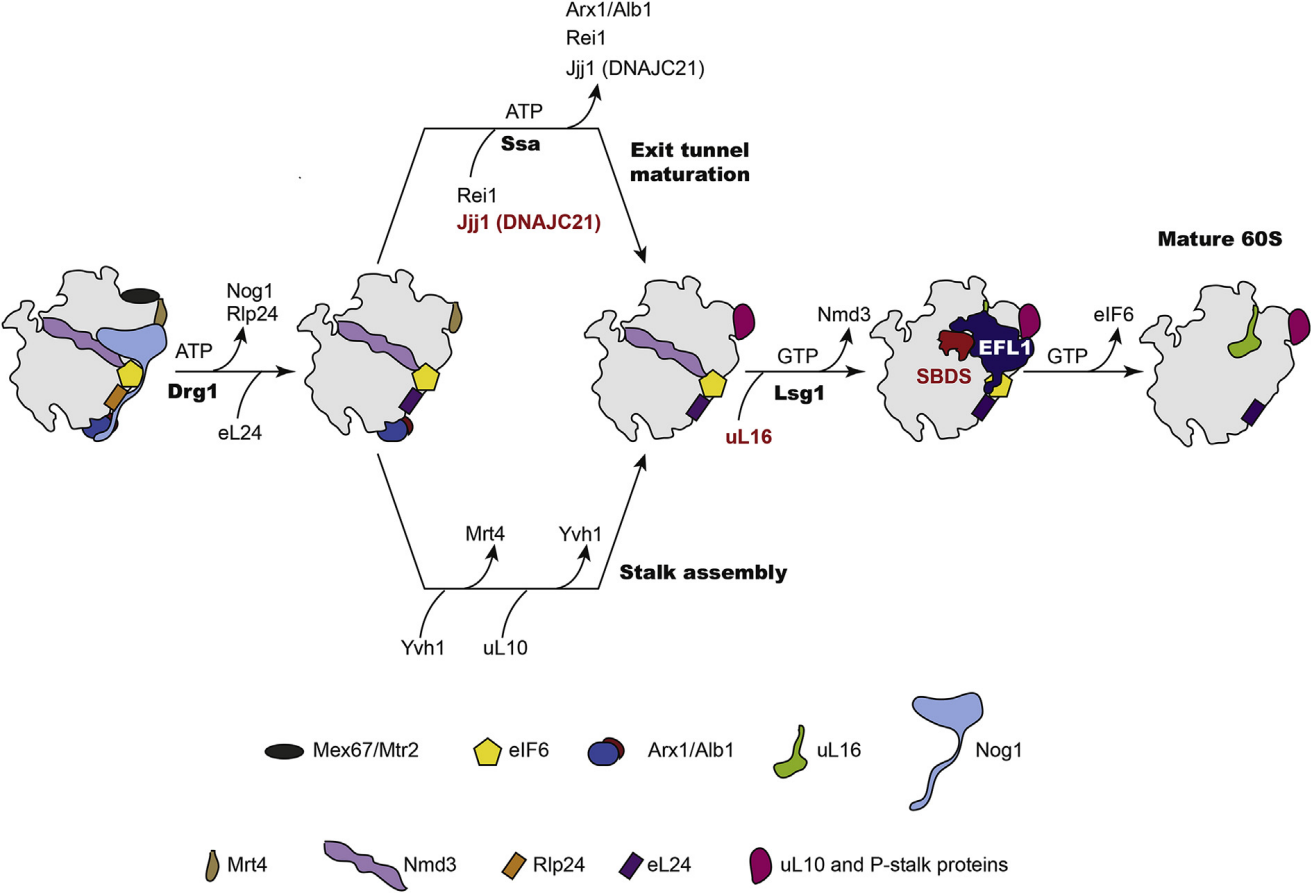
Schematic overview of cytoplasmic 60S subunit assembly. The AAA-ATPase Drg1 promotes release of the GTPase Nog1 and the exchange of Rlp24 for Rpl24 that in turn recruits Rei1. The J-protein Jjj1 (human DNAJC21) cooperates with the ATP-dependent Hsp70 chaperone SSa to release the export receptor Arx1 that lies close to the polypeptide exit tunnel. In a parallel pathway, Yvh1 facilitates the exchange of Mrt4 with P0 to assemble the ribosome stalk. Recruitment of uL16 in concert with the GTPase Lsg1 promotes release of the export adaptor Nmd3. Finally, release of eIF6 from the subunit joining face by SBDS and the EFL1 GTPase licences entry of the mature subunit into the pool of actively translating ribosomes. Proteins mutated in human disease are highlighted in red. DNAJC21 and SBDS are mutated in Shwachman-Diamond syndrome; uL16 is mutated in paediatric T-ALL.

**Fig. 2:**
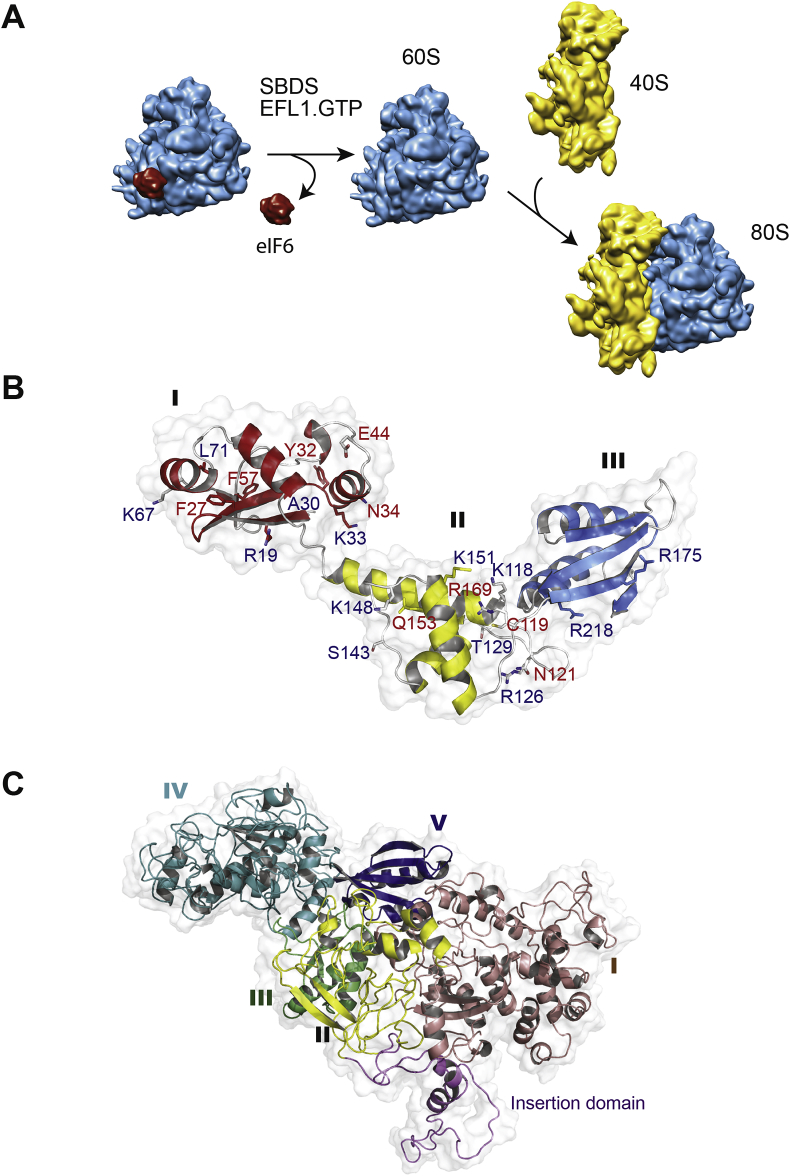
SBDS and the EFL1 GTPase cooperate to catalyse eIF6 eviction. **(A)** Schematic showing that SBDS cooperates with the GTPase EFL1 in the cytoplasm to catalyse release of the anti-association factor eIF6 from the 60S subunit joining face to allow subunit joining and the formation of translation-competent 80S ribosomes. 40S subunit is coloured yellow, 60S subunit is cyan, eIF6 is red. **(B)** Ribbon representation of the human SBDS NMR structure. Domain I is coloured red, domain II is yellow, domain III is blue and loops are coloured grey. SDS-associated mutations modify surface epitopes (red text) or affect protein stability (blue text). (**C**) Ribbon representation of the atomic model of human EFL1 (Weis et al., 2015), with domain I coloured violet, domain II yellow, domain III green, domain IV cyan and domain V blue.

The majority of the transiently interacting assembly factors are released prior to nuclear export. Completion of large subunit maturation in the cytoplasm involves recruitment of the last remaining ribosomal proteins, assembly of the peptidyl transferase centre (PTC), removal the final assembly factors and release of the nascent 60S subunit into the actively translating pool of ribosomes (Fig. 1). The AAA-ATPase Drg1 initiates cytoplasmic maturation of the 60S subunit by removing Rlp24 (the placeholder for ribosomal protein eL24 (Ban et al., 2014)) and the GTPase Nog1 (Kappel et al., 2012, Pertschy et al., 2007). Recruitment of eL24 promotes the binding of Rei1, which cooperates with the J-protein Jjj1 and its Hsp70 co-chaperone Ssa to release Arx1 from the exit tunnel (Demoinet et al., 2007, Hung and Johnson, 2006, Lebreton et al., 2006, Meyer et al., 2007, Meyer et al., 2010). In a parallel pathway, the P-stalk is generated by Yvh1-dependent replacement of the placeholder protein Mrt4 with the stalk protein uL10 (Kemmler et al., 2009, Lo et al., 2009, Rodriguez-Mateos et al., 2009). The final two maturation steps are the removal of Nmd3 by the concerted action of the GTPase Lsg1 and ribosomal protein uL16 (Hedges et al., 2005, West et al., 2005) and release of Tif6 (mammalian eIF6) by elongation factor-like GTPase 1 (Efl1, human EFL1) and its cofactor Sdo1 (the yeast SBDS orthologue) (Becam et al., 2001, Menne et al., 2007, Senger et al., 2001) (Fig. 2A–C).

The eIF6 protein is a nuclear shuttling assembly factor that is highly conserved in archaea and all eukaryotes. Originally identified as an anti-association factor that inhibits joining of the 40S and 60S ribosomal subunits in wheat germ extracts (Russell and Spremulli, 1978, Russell and Spremulli, 1979, Valenzuela et al., 1982), eIF6 is essential in yeast (Si and Maitra, 1999) and mammals (Sanvito et al., 1999). The eIF6 protein is required in the nucleus for ITS2 pre-rRNA processing and exported with the nascent pre-60S subunit to the cytoplasm where it must be removed to permit joining of the nascent 40S and 60S ribosomal subunits, thereby activating translation (Ceci et al., 2003). The eIF6 protein has a conserved pentein fold consisting of five copies of a repeating α/β subdomain of about 45 residues with an internal five-fold axis of pseudosymmetry (Groft et al., 2000). Removal of eIF6 is a prerequisite for the translational activation of ribosomes because it binds to the sarcin-ricin loop (SRL) and to ribosomal proteins uL3, uL14 and eL24 on the intersubunit face of the large ribosomal subunit, thereby physically impeding ribosomal subunit joining (Gartmann et al., 2010, Klinge et al., 2012b, Weis et al., 2015).

## 4 SBDS cooperates with the GTPase EFL1 to catalyse eIF6 removal

What is the evidence that the SBDS and the GTPase EFL1 function in the removal of eIF6 from nascent 60S ribosomal subunits? A suppressor screen in yeast revealed that gain-of-function *TIF6* alleles could rescue the fitness defect of Efl1 deleted cells (Becam et al., 2001, Senger et al., 2001). Furthermore, genome-wide synthetic genetic array mapping revealed that multiple gain-of-function *TIF6* alleles could suppress the defective growth, attenuated translation, defective pre-60S nuclear export and cytoplasmic mislocalisation of Tif6 in cells deleted for either Sdo1, the GTPase Efl1 or both Sdo1 and Efl1 (Menne et al., 2007). Remarkably, all 28 independent amino acids targeted by gain-of-function suppressor mutations mapped to a surface of Tif6 that was predicted (Menne et al., 2007) and later confirmed by X-ray crystallography (Klinge et al., 2012a) to interact with the 60S ribosomal subunit. Taken together, these data provided strong genetic evidence that Tif6 is the direct target for the concerted action of both Sdo1 and Efl1.

The next step was to show biochemically that eIF6 is indeed the direct target of both SBDS and EFL1. To develop an assay for eIF6 release, native cytoplasmic pre-60S ribosome particles enriched in bound eIF6 were purified from Sbds-deleted mouse liver cells (Finch et al., 2011). Deletion of Sbds caused a marked ribosomal subunit-joining defect due to retention of eIF6 on the intersubunit face of late pre-60S particles, impeding the assembly of actively translating 80S ribosomes. In the presence of GTP, recombinant human SBDS and EFL1 co-catalysed the release of eIF6 from the purified mammalian pre-60S particles by a mechanism dependent on GTP binding and hydrolysis by EFL1. Although the 60S subunit strongly activated EFL1 GTPase activity in vitro, the SBDS protein further enhanced this biochemical activity. However, the use of informative disease-associated SBDS mutants revealed that the key biochemical function of SBDS is to couple GTP hydrolysis by EFL1 to eIF6 removal from the nascent 60S subunit.

The genetically tractable ancient eukaryote *Dictyostelium discoideum* was exploited to test the generality of the hypothesis that SBDS has a highly conserved role in maturation of the 60S ribosomal subunit (Wong et al., 2011). As the *Dictyostelium* SBDS orthologue is essential at the cellular level, a temperature-sensitive intein was introduced into the endogenous *Dictyostelium sbdS* gene by homologous recombination. The intein spliced itself out from the SBDS protein at the permissive temperature, but at the restrictive temperature functional SBDS protein levels were dramatically reduced, severely impairing ribosomal subunit joining due to defective release and recycling of eIF6 from the 60S subunit joining face. Importantly, wild type human SBDS, but not disease-associated variants, fully complemented the growth and ribosome assembly defects observed in the mutant *Dictyostelium* cells. Reciprocal co-immunoprecipitation experiments revealed that the endogenous *Dictyostelium* SBDS and EFL1 proteins directly interact on the nascent 60S ribosomal subunit in vivo. In biochemical experiments, human SBDS and EFL1 catalysed GTP-dependent dissociation of the endogenous eIF6 protein from purified *Dictyostelium* pre-60S ribosomal subunits. Finally, SDS patient-derived lymphoblast cell lines harboured ribosomal subunit joining defects whose magnitude was inversely proportional to the level of residual SBDS protein expression. Consistent with these data, depletion of eIF6 improved the subunit-joining defect in SDS patient cells (Burwick et al., 2012). In conclusion, genetic and biochemical data from *Dicytostelium*, yeast, mice and SDS patient-derived cells strongly support the hypothesis that SDS is a human ribosomopathy caused by the impaired release and recycling of eIF6 from late cytoplasmic 60S ribosomal subunits.

## 5 Functional significance of eIF6 phosphorylation

In mammalian cells, an alternative model posits that RACK1 (receptor for activated C kinase 1) recruits PKCβII to the ribosome to promote eIF6 eviction by phosphorylating eIF6 residue S235 (Ceci et al., 2003). However, genetic data in mammalian cells and in yeast do not support such a model. First, PKCβ null mutant mice are viable and grow normally (Bansode et al., 2008). Secondly, in yeast, the phylogenetically conserved Tif6 residues 1–224 are sufficient for Tif6 function and recycling in vivo (Weis et al., 2015). Thirdly, although phosphorylation of Tif6 residues S174 and S175 by the casein kinase 1 homolog Hrr25 was proposed to be essential for Tif6 recycling (Ray et al., 2008), cells carrying a *TIF6-S174A*, *S175A* double mutant allele as the sole copy of the *TIF6* gene had no loss in fitness compared with wild type cells (Weis et al., 2015). Thus, clarification of the functional significance of eIF6 phosphorylation in vivo is required in both yeast and mammals.

## 6 Intrinsic flexibility of the SBDS protein

To better understand the function of the SBDS protein at a molecular level, its structure and dynamic properties have been investigated in detail. The SBDS protein is widely expressed and highly conserved, with orthologues in prokaryotes and all eukaryotes, but not in eubacteria (Boocock et al., 2003). X-ray crystallography (Ng et al., 2009, Savchenko et al., 2005, Shammas et al., 2005) and NMR spectroscopy (de Oliveira et al., 2010, Finch et al., 2011) revealed a conserved tripartite structure for the SBDS protein (Fig. 2B). The fold of the SBDS N-terminal or FYSH (Fungal, Yhr087w, Shwachman) domain (residues S2—S96) is shared with a single-domain yeast protein (Yhr087w) (Shammas et al., 2005) that appears to be involved in translational regulation (Gomar-Alba et al., 2012). The SBDS central domain (residues D97-A170) comprises a three-helical, right-handed twisted bundle, while the C-terminal domain (residues H171-E250) has a ferredoxin-like fold with striking structural similarity to domain V of elongation factor 2 (and EFL1) (Shammas et al., 2005). Overall, the SBDS protein is similar in size and shape to tRNA (Ng et al., 2009) and to ribosome recycling factor (RRF) (Finch et al., 2011).

EFL1 is a cytoplasmic GTPase that is homologous to prokaryotic elongation factor G (EF-G, EF2 in archaea) and the ribosomal translocase EF-2 in eukaryotes. Like EF-G, EFL1 has a five-domain architecture (Weis et al., 2015), but it is distinguished from EF-G by a loop insertion of variable length within domain II (Fig. 2C). Interestingly, while orthologues of EF2, SBDS and eIF6 are found in archaea, EFL1 is absent, suggesting that EF2 has dual roles in biogenesis (eIF6 release) and in translation in archaea. The structural homology between EFL1 and the ribosomal translocase EF-G, coupled to the ability of EFL1 to compete out the inhibition of ribosome-associated EF-2 GTPase activity by fusidic acid (Graindorge et al., 2005), suggested that EFL1 and EF-G likely bind to a similar position at the GTPase centre of the ribosome and that SBDS might transiently recruit EFL1 to the GTPase centre of the ribosome (Menne et al., 2007).

The SBDS protein is highly dynamic and intrinsically flexible. X-ray crystallography revealed two distinct conformations for *Methanothermobacter thermautotropicus* SBDS (Ng et al., 2009) and a third for *Archaeoglobus fulgidus* SBDS (Shammas et al., 2005). NMR relaxation experiments revealed the dynamic motion of individual SBDS protein domains and fast time-scale internal mobility that is conserved between human and yeast (Finch et al., 2011). The N-terminal domain of the SBDS protein tumbles semi-independently relative to domains II and III through a hinge that is centred within an N-terminal helix (α5) that itself has a high degree of internal flexibility. Interdomain motion and flexibility of the SBDS protein in the context of the ribosome was subsequently revealed by single-particle cryo-EM studies (Weis et al., 2015).

## 7 SBDS is an allosteric regulator of the EFL1 GTPase

How precisely do SBDS and EFL1 cooperate to catalyse the removal of eIF6 from nascent 60S ribosomal subunits? The recent developments in single particle cryo-EM (Frank, 2017) provided an important tool to investigate these questions. Human SBDS genetically complements *Dictyostelium* cells lacking the endogenous SBDS protein and in biochemical assays, human SBDS and EFL1 catalyse the removal of endogenous eIF6 from purified *Dictyostelium* pre-60S subunits in the presence of GTP (Wong et al., 2011). This provided a rationale for using a heterologous mixture of *Dictyostelium* eIF6-bound 60S subunits (purified from a mutant expressing a dominant-negative SBDS protein variant), human SBDS, human EFL1 and non-hydrolysable GTP analogue (GMPPCP) as the substrate for single-particle cryo-EM experiments (Weis et al., 2015).

**Fig. 3:**
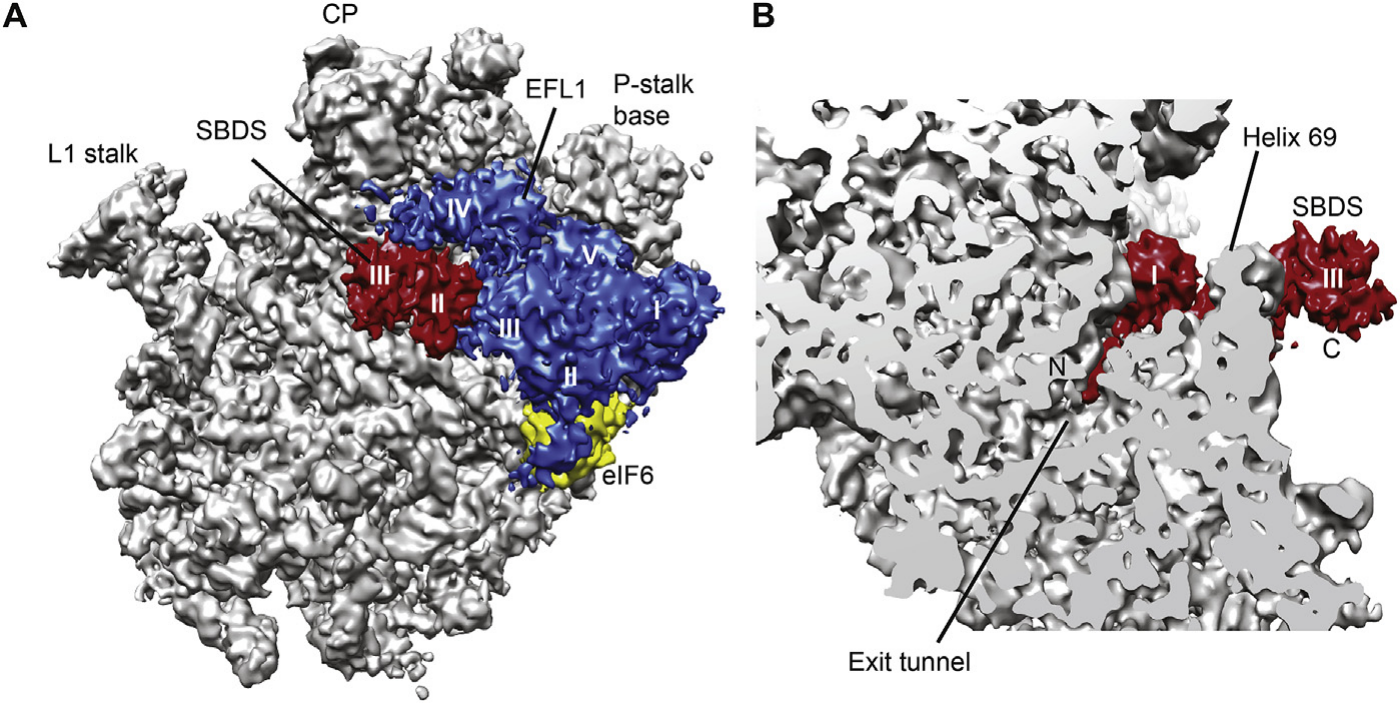
SBDS and EFL1 proofread the structural and functional integrity of the nascent 60S subunit. (**A**) Crown view and (**B**) transverse section of the cryo-EM maps of the 60S-eIF6-SBDS-EFL1 complex. The 60S subunit is coloured grey, SBDS is red, EFL1 is blue and eIF6 is yellow. SBDS domains II and III and EFL1 domains I-V are shown. CP, central protruberance.

The structures of three independent complexes (eIF6-SBDS, eIF6-SBDS-EFL1, and SBDS-EFL1 bound to the 60S subunit) were determined, revealing that the SBDS protein binds to the ribosomal P-site on the intersubunit face of the 60S subunit (Fig. 3A and B) in close proximity to a conserved essential internal loop of ribosomal protein uL16 that is targeted by mutations in paediatric T-cell acute lymphoblastic leukaemia (T-ALL) (De Keersmaecker et al., 2013). This is interesting, because it reveals a direct physical link on the ribosome between two proteins (SBDS and uL16) mutated in inherited (SDS) and sporadic (T-ALL) forms of leukaemia. The N-terminus of the SBDS protein (residues S2–V15) interacts with components of the PTC, with the six most N-terminal residues extending into the upper part of the ribosomal peptide exit tunnel (Fig. 3B). SBDS domain III interacts with the sarcin-ricin loop (SRL) and the P-stalk base in a similar manner to domain V of prokaryotic EF-G and yeast EF-2. Thus, SBDS effectively interrogates the key active sites of the 60S subunit, including the P-site, PTC, the entrance to the polypeptide exit tunnel and the binding site for the translational GTPases.

Upon EFL1 binding, SBDS undergoes a dramatic conformational rearrangement in which domain III rotates by 180° away from the P-stalk base (closed state) to interact with helix 69 (open state), while SBDS domain I remains anchored in the P-site. The conformational changes in SBDS visualised by cryo-EM are consistent with the global dynamic motion of the SBDS protein observed off the ribosome using NMR spectroscopy (Finch et al., 2011). Interestingly, the closely related folds of SBDS domain III and EFL1 domain V permit both proteins to bind consecutively to a common site on the ribosome at base of the P-stalk. EFL1 binds to the GTPase centre, interacting predominantly with SBDS and eIF6. The conformation of EFL1 differs significantly in the presence and absence of bound eIF6 due to a pivoting movement of EFL1 domains I–II and IV around domains III and V. Importantly, the more extended (accommodated) EFL1 conformation competes with eIF6 for an overlapping binding site on the SRL and is incompatible with simultaneous binding of eIF6.

## 8 Proposed mechanism of eIF6 release

Based on structural, biochemical, and genetic data, a conformational switching mechanism for eIF6 release by SBDS and EFL1 has been proposed (Weis et al., 2015). Although the precise timing remains unclear, SBDS is likely recruited to a late cytoplasmic eIF6-bound pre-60S particle following assembly of the P-stalk base, loading of uL16 and the removal of Nmd3. SBDS initially binds between the P-site and the base of the P-stalk in (the ‘closed’ state), while EFL1 binds to the GTPase centre of the 60S particle in a low-affinity GTP-bound state. Domain V of EFL1 competes with SBDS domain III for an overlapping binding site at the base of the P-stalk, promoting a 180-degree rotational displacement of SBDS domain III away from the P-stalk base toward helix 69 (open state). The conformational equilibrium of EFL1 then shifts towards a high-affinity “accommodated” state that efficiently competes with eIF6 for an overlapping binding site on the SRL. EFL1 GTP hydrolysis is activated by the SRL, shifting the EFL1 conformational equilibrium towards a low-affinity binding state that facilitates EFL1 dissociation from the 60S subunit. The structural data support the concept of an allosteric cascade in which large-scale movements in SBDS and EFL1 link the conserved P-site loop of uL16 with eIF6. The proposed mechanism of eIF6 eviction is reminiscent of bacterial ribosome recycling by RRF (homologous to SBDS) and EF-G (homologous to EFL1).

In summary, SBDS functions as a multi-tasking 60S subunit assembly factor that 1) promotes allosteric conformational switching of the EFL1 GTPase and 2) protects and proofreads the P-site and the base of the P-stalk. SBDS domain I protects and proofreads the structural integrity of the peptide exit tunnel and the PTC; domains I and II couple dynamic interdomain motion to EFL1 conformational switching; SBDS domain III interrogates the translational GTPase-binding site at the P-stalk base in the closed state.

Is eIF6 release the final step in 60S subunit maturation? The order of release of the assembly factors Nmd3 and eIF6 has been controversial. Genetic data suggested that Nmd3 is removed after eIF6 (Lo et al., 2010). However, the structure of late pre-60S particles from *Dictyostelium* cells overexpressing a dominant negative SBDS variant reflected that of a mature 60S subunit enriched in eIF6 but not Nmd3, with uL16 already incorporated. Furthermore, yeast cells carrying the T-ALL-associated *uL16-H123P* allele as the sole copy of uL16 lacked detectable expression of uL16 that is required both for Nmd3 release and for Sdo1 recruitment to the 60S subunit in vivo (Weis et al., 2015). Taken together, these data support a model in which SBDS is recruited to the pre-60S subunit after uL16 binding and the removal of Nmd3. Further support for this revised model comes from cryo-EM structural analysis showing that Nmd3 physically overlaps with and prevents access of SBDS to the P-site (Ma et al., 2017, Malyutin et al., 2017). A central domain of Nmd3 that is structurally homologous to eIF5A binds in the E site of the ribosome, pulling the L1 stalk into a closed position. The junction between the N-terminal and eL22-like domains of Nmd3 occupies the P site, while the N-terminal domain extends towards the SRL, directly interacting with eIF6. Finally, complementary genetic and biochemical studies have shown that the T-ALL-associated *uL16-R98S* missense mutant disrupts Nmd3 release, thereby blocking the access of Sdo1 to the P-site that in turn reduces Efl1 binding and the subsequent removal of eIF6 (Patchett et al., 2017).

Consistent with an essential role during late cytoplasmic maturation of the 60S ribosomal subunit maturation, the SBDS protein has a conserved cytoplasmic distribution protein in multiple species, including human cells (Ball et al., 2009, Finch et al., 2011, Menne et al., 2007, Wong et al., 2011). In addition, the integration of uL16 into nascent pre-60S ribosomal subunits in the cytoplasm is an essential prerequisite for SBDS recruitment in vivo (Weis et al., 2015). The functional significance of the proposed nucleolar enrichment of SBDS still remains unclear (Austin et al., 2005).

## 9 Interpreting disease-associated SBDS variants

NMR spectroscopy was used to interrogate the impact of 29 disease-associated missense variants on the structural integrity of the SBDS protein and classify them into two discrete groups: those affecting the overall stability or fold of SBDS and those that alter surface epitopes without altering the stability or fold of the protein (Finch et al., 2011). The cryo-EM structures of SBDS bound to the nascent 60S subunit reveal that pathological missense variants disrupt key contacts required for SBDS binding to the rRNA of the 60S subunit or perturb interactions that stabilise important conformational states (Weis et al., 2015). Several mutations (Y32C, F27L, F57L, C84R) destabilise the fold of SBDS domain I, disrupting the interaction with the ribosomal P-site. Residue K67 (SBDS domain I) likely makes a key electrostatic interaction with the P loop rRNA, while residues K151 (N-terminus of helix α7, domain II) and R218 (helix α9, domain III) are important for stabilising the open conformation of SBDS through interaction with the tip of helix 69. Loss of function mutations (*Δ94-95* or *D97-K98delinsEVQVS*) alter the length of the flexible linker between domains I and II, highlighting a key role for the domain I-II linker in allowing conformational switching of the SBDS protein on the 60S subunit.

## 10 SDS and the quality control of ribosome assembly

Why use such a complex strategy to evict eIF6 during the final step in maturation of the large ribosomal subunit? One explanation is that it provides a mechanism to couple eIF6 release to a final quality control assessment of the structural integrity of key functional sites on the nascent 60S subunit. Together, SBDS and EFL1 proofread the integrity of the polypeptide exit tunnel, the P-site, the GTPase centre, the base of the P-stalk and the SRL. The six N-terminal residues of SBDS insert into the proximal end of the exit tunnel, while the C-terminus of the zinc finger assembly factor Rei1 probes the distal part of the tunnel (Greber et al., 2016). Thus, Rei1 and SBDS between them interrogate the integrity of the entire length of the polypeptide exit tunnel during 60S assembly. Finally, competition between EFL1 and eIF6 for an overlapping binding site on the SRL provides an elegant mechanism to couple a final quality control assessment of the structural and functional integrity of the SRL to the last step in the EFL1 catalytic cycle, the activation of GTP hydrolysis. Hence, SBDS and EFL1 emerge as key gatekeepers that license the entry of functionally competent ribosomes into the actively translating pool.

## 11 Does SBDS function independently of the ribosome?

A fundamental conserved role for the SBDS protein in late cytoplasmic maturation of the large ribosomal subunit has been established using a wide range of genetic, biochemical and structural approaches in multiple independent species. Nevertheless, an increasing array of functions has been proposed for SBDS, including roles in rRNA processing (Koonin et al., 2001, Rujkijyanont et al., 2009, Savchenko et al., 2005, Wu et al., 2002), chemotaxis (Wessels et al., 2006), mitotic spindle stabilisation (Austin et al., 2008), cellular stress responses (Ball et al., 2009), Rac2-mediated monocyte migration (Leung et al., 2011), Fas ligand-induced apoptosis (Rujkijyanont et al., 2008), the regulation of lysosome homeostasis (Vitiello et al., 2010), mitochondrial function (Henson et al., 2013). However, in the absence of compelling evidence supporting a direct biochemical function for SBDS in any of these processes, the most logical explanation is that all these phenotypes are downstream secondary consequences of a primary defect in ribosome biogenesis and attenuated translation. This conclusion is strongly supported by the identification of mutations in the 60S ribosomal subunit assembly factor *DNAJC21* in SDS patients who are negative for mutations in the *SBDS* gene.

## 12 DNAJC21: a new player in Shwachman-Diamond syndrome

Biallelic mutations in the *DNAJC21* gene (GenBank: NM_001012339.2) have recently been identified in eight individuals with SDS who were negative for mutations in the *SBDS* gene. Four individuals presented similarly with bone marrow failure, intra-uterine growth retardation and/or short stature, dental abnormalities, hyperkeratosis, skin pigmentation and retinal dystrophy, one developing acute megakaryocytic leukaemia at 12 years of age (Tummala et al., 2016). The *DNAJC21* mutations included two homozygous nonsense variants (c.517C > T, p.R173∗; c.793G > T, p.E265∗), a splice variant (c.983+1G > T) and a missense mutation (c.94C > G, p.P32A). Another four patients from three unrelated families who had been diagnosed clinically with SDS carried biallelic mutations in *DNAJC21*, including two nonsense mutations (p.Q174*; p.V148Kfs*30) and a missense variant (c.100A > G, p.K34E) (Dhanraj et al., 2017). Interestingly, two of these individuals also had retinal dystrophy.

**Fig. 4:**
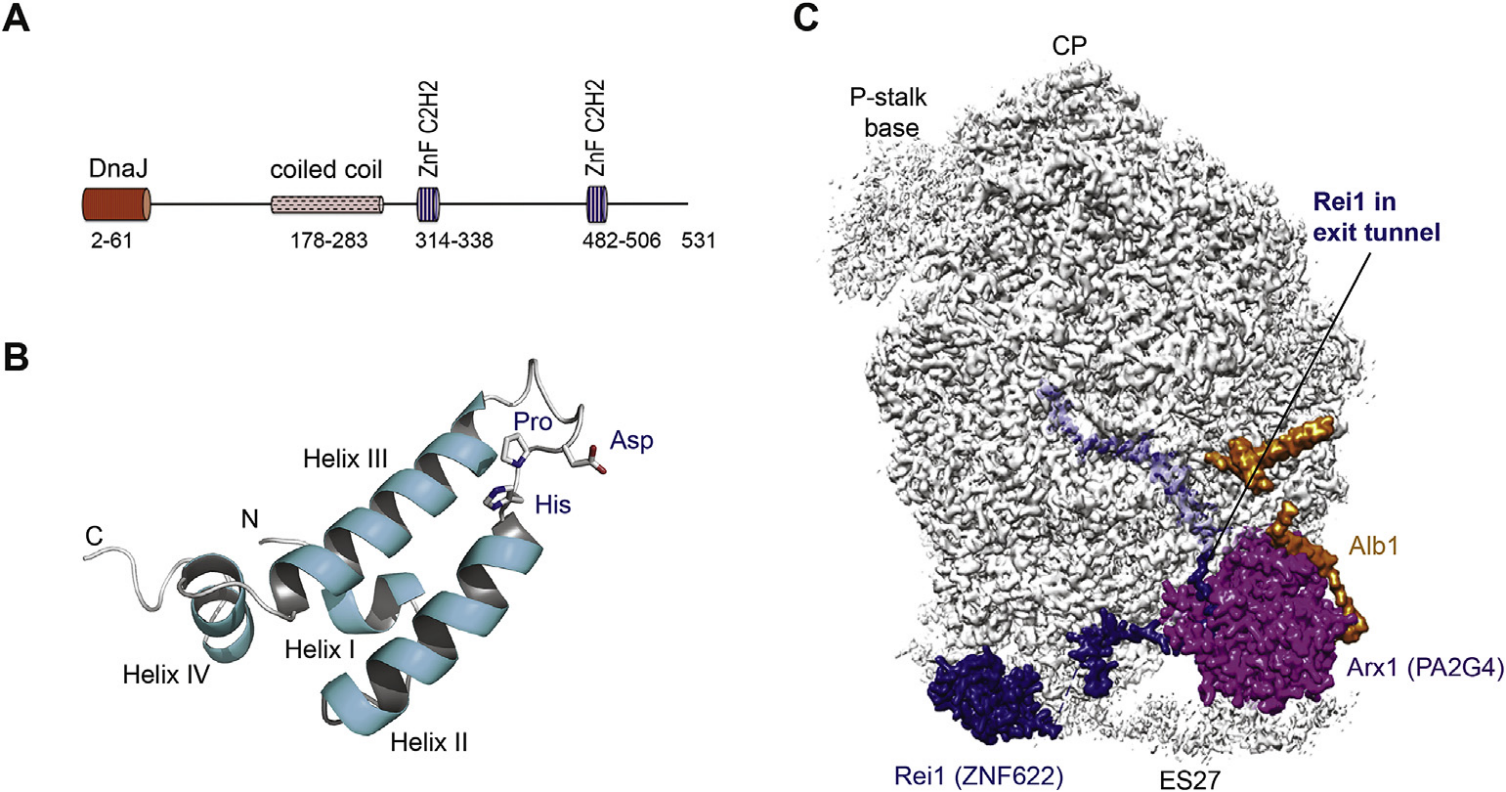
The J-protein DNAJC21 facilitates release of the export receptor Arx1 (human PA2G4) from the pre-60S ribosomal subunit. (A) Schematic representation of the domain structure of human DNAJC21 (NP_001335349). ZnF, zinc finger. (B) Ribbon representation of the NMR structure of the J-domain (residues 2–108) from *E. coli* DnaJ protein (pdb 1xbl) (Pellecchia et al., 1996). J-domains comprise four α-helices. The invariant His, Pro, Asp (HPD) tripeptide in the loop between helices II and III is critical to stimulate the ATPase activity and in vivo function of its cognate cochaperone heat shock 70 kDa protein. (C) Visualising the 60S-bound assembly factors Arx1 (human PA2G4), Rei1 (human ZNF622) and Alb1 near the polypeptide exit tunnel (pdb 5APO) (Greber et al., 2012). The Arx1 protein shields the polypeptide exit tunnel, which is deeply probed by the C-terminus of the Rei1 protein. Alb1 likely modulates the affinity of Arx1 binding to the 60S subunit.

DNAJC21 belongs to the family of J proteins (named after the founding member, *E. coli* DnaJ) that interact with client heat shock protein 70 (Hsp70) chaperones to stimulate their ATPase activity (Fig. 4A–C). By definition, J proteins contain a highly conserved ∼70 amino acid J domain, comprising four α-helices with a conserved His, Pro, Asp (HPD) tripeptide that lies within a helical hairpin between helices II and IV (Pellecchia et al., 1996). The HPD motif lies at the interaction interface with the Hsp70 client protein and is critical for stimulating Hsp70 ATPase activity (Greene et al., 1998, Jiang et al., 2007). DNAJC21 is a ubiquitously expressed 531 amino acid protein that contains a highly conserved N-terminal J domain and two zinc finger (C2H2 type) domains. The yeast DNAJC21 homologue, Jjj1, is a cytosolic J protein that functions together with the cytoplasmic zinc-finger protein Rei1 (human zinc finger protein 622, ZNF622) to stimulate the ATPase activity of the Hsp70 chaperone protein Ssa (human heat shock 70 kDa protein 8 (HSPA8)), thereby promoting the release and recycling of the nuclear export factor Arx1 (human proliferation-associated protein 2G4 (PA2G4), also known as Erbb3-binding protein 1 (EBP1) and IRES-specific cellular transacting factor 45 (ITAF45)) that binds near the ribosome peptide exit tunnel (Demoinet et al., 2007, Hung and Johnson, 2006, Lebreton et al., 2006, Meyer et al., 2007, Meyer et al., 2010). The role of human DNAJC21 in the release and nuclear recycling of PA2G4 appears to be conserved (Tummala et al., 2016). The efficient removal of yeast Tif6 depends on the prior release of Arx1 by Jjj1-Rei1-Ssa (Lebreton et al., 2006, Lo et al., 2010), linking Jjj1 and Sdo1-Efl1 in a common pathway involved in late cytoplasmic 60S ribosomal subunit maturation. How the removal of Arx1 regulates Tif6 release remains unclear, but the maturation steps at the exit tunnel are clearly coordinated with events at the 60S subunit interface.

How do the J-proteins Jjj1 and the Hsp70-like ATPase Ssa remove Arx1 and Rei1 from the nascent 60S particle? Structural studies suggest that Jjj1 binds near the Arx1-Rei1 interaction site close to a Rei1 linker which projects from the polypeptide exit tunnel (Greber et al., 2016). Jjj1 likely recruits and activates Ssa at this site, promoting ATP-dependent removal of both Rei1 and Arx1. However, further experiments are required to establish whether Ssa releases Arx1 by disrupting the interaction between Arx1 and Rei1 or if Arx1 and Rei1 are simultaneously released after Ssa extracts Rei1 from the exit tunnel. Together, Jjj1, Arx1 and Rei1 likely proofread the integrity of the polypeptide exit tunnel and the nearby binding platform involved in protein biogenesis. Arx1 may interrogate the integrity of the binding sites for the signal recognition particle and the translocon, two key factors involved in co-translational membrane protein targeting, while Rei1 proofreads the ability of the exit tunnel exit tunnel to conduct nascent polypeptides. Finally, it is possible that Jjj1 checks the integrity of the binding site for the RAC chaperone complex (Gautschi et al., 2001) that contains the structurally related J-protein Zuo1 (human ZRF1) (Yan et al., 1998). The RAC heterodimer stimulates the ATPase activity of the ribosome-bound Ssb through the J-domain of Zuo1 (Huang et al., 2005) and interacts with nascent polypeptide chain to facilitate de novo protein folding. Intriguingly then, both of the ribosome assembly factors mutated in SDS (SBDS and DNAJC21) appear to function in proofreading the structural integrity of the maturing 60S subunit.

How do SDS-associated mutations in DNAJC21 affect the function of the protein? *DNAJC21* transcripts containing nonsense and predicted frameshift mutations likely undergo nonsense-mediated mRNA decay, causing severe reduction or loss of DNAJC21 protein expression in patient samples (Dhanraj et al., 2017, Tummala et al., 2016). The missense variant p.P32A likely alters the fold of the critical J domain HPD motif, disrupting the interaction with HSPA8 and stimulation of its ATPase activity. The p.K34E missense variant reverses the surface charge of a key amino acid adjacent to the HPD motif and also likely disrupts the interaction with HSPA8. Interestingly, individuals lacking functional DNAJC21 are viable, whereas biallelic *SBDS* null mutations do not appear to be compatible with life.

## 13 Homozygous *EFL1* mutations phenocopy Shwachman-Diamond syndrome

Four individuals homozygous for a p.R1095Q variant and two individuals homozygous for a p.M882K variant in the EFL1 GTPase were recently identified by exome sequencing (Stepensky et al., 2017). Hypomorphic *EFL1* mutations might be predicted to phenocopy SDS clinically, as EFL1 and SBDS functionally cooperate to catalyse eIF6 release during the final cytoplasmic step in 60S ribosomal subunit maturation (Finch et al., 2011, Menne et al., 2007, Weis et al., 2015, Wong et al., 2011). Interestingly, the clinical phenotype does indeed have features of classical SDS, including pancytopenia, exocrine pancreatic insufficiency and skeletal defects. This raises an interesting point: do we designate the *EFL1*-mutated clinical phenotypes as SDS or SDS-like, the term used by Stepensky et al.? This will be an interesting topic for future discussion. Importantly, the potential functional impact of the reported EFL1 missense variants on human ribosome assembly was not assessed and the introduction of the corresponding disease-related mutations in yeast had no significant impact on cell fitness. Thus, further work is required to validate the functional consequences of the reported human EFL1 mutations on 60S subunit maturation.

## 14 Tissue specificity in Shwachman-Diamond syndrome

The diverse phenotypes associated with the human ribosomopathies raise a key conundrum: how do mutations that perturb the ubiquitous process of ribosome assembly cause such tissue specific defects? Consistent with a fundamental conserved role for the SBDS protein in 60S subunit maturation and ribosomal subunit joining, global translation is reduced in Sdo1-deficient yeast cells (Menne et al., 2007), in mouse embryonic fibroblasts expressing the disease-related *Sbds-R126T* missense mutation (Calamita et al., 2017) and in human kidney 293 cells depleted of SBDS by siRNA (Ball et al., 2009). The sensitivity of adult mouse liver cells to Sbds deletion may reflect the high rates of global protein translation in this organ (Finch et al., 2011). The reduced availability of ribosomes may also selectively perturb the translation of specific mRNA subsets containing upstream open reading frames (uORFs) or complex 5′ untranslated regions (UTRs). Indeed, the translation of uORF-containing mRNAs encoding critical regulators of granulocytic differentiation (C/EBPα and -β) is perturbed in SBDS-deficient cells (In et al., 2016). Perturbed translation of a subset of homeobox mRNAs containing complex 5’ UTRs in mice carrying mutations of the *Rpl38* gene cause tissue-specific patterning defects with marked homeotic transformations of the axial skeleton. Thus, impaired global translation and reduced tissue-specific translation of complex mRNAs may both contribute to the SDS phenotype as a consequence of the reduced availability of functional 60S subunits entering the actively translating pool.

An additional tier of translational control involves the dynamic recycling of post-termination ribosomes from the 3′UTR of mRNAs by the ribosome rescue factors PELO, HBS1L and ABCE1 (Mills et al., 2017). It is possible that the expression levels of these rescue factors may modulate the sensitivity of specific cell types when the actively translating pool of ribosomes is limiting.

The attenuation of global translation evokes a range of molecular responses downstream of p53 activation, resulting in completely different outcomes depending on the specific tissue and developmental timing. Conditional disruption of the *Sbds* gene in the pancreas induced atrophy of post-natal acinar cells due to p53-dependent loss of digestive enzyme synthesis and the induction of senescence (Tourlakis et al., 2015). Pancreatic acinar cells appear to be more susceptible to the increase demand for protein synthesis in the post-natal period around the time of weaning. In contrast, p53-dependent apoptotic cell death was induced in neuronal cells. The diversity of tissue-specific responses to impaired ribosome biogenesis is further highlighted by genetic studies in zebrafish (*Danio rerio*). Depletion of ribosomal protein rpl3 or the 60S subunit assembly factors sbds, pescadillo (Provost et al., 2012) and nol9 (an essential conserved polynucleotide 5’ kinase involved in ITS2 pre-rRNA processing) (Bielczyk-Maczynska et al., 2015) caused a p53-independent proliferation defect in *ptf1a*-expressing pancreatic progenitor cells. By contrast, the proliferation defects in haematopoietic stem cells in *nol9* mutant zebrafish were p53-dependent. In a further example, p53-dependent disruption of biliary tract development and function was evoked by morpholino knockdown of the zebrafish CIRHIN homologue (Wilkins et al., 2013).

In summary, the tissue proclivity of the SDS phenotype likely reflects the consequences of the reduced availability of actively translating ribosomes (either through reduced subunit joining or impaired ribosome recycling), particularly when the proliferative rates of affected tissues are high during specific stages of developmental. Perturbation of both global and tissue-specific mRNA translation (uORFs and complex 5′UTRs) may also play a role. Tissue-specific stress responses to impaired translation and the downstream consequences of p53 activation also influence the phenotypic outcome. Elucidating the mechanisms underlying the differing molecular responses to p53 activation is a key challenge for the future.

## 15 Dameshek's riddle and Shwachman-Diamond syndrome

The poorly understood mechanism of clonal progression to MDS/AML in SDS is encapsulated by Dameshek's riddle: how does attenuated translation and a hypoproliferative state (bone marrow failure) promote the transition to a hyperproliferative disorder (leukaemia) (Dameshek, 1967)? Haematopoietic stem cells appear to be particularly sensitive to altered (either increased or decreased) levels of protein synthesis. For example, Pten deficiency in murine haematopoietic stem and progenitor cells (HSPCs) promoted leukaemia partly by increasing protein synthesis (Signer et al., 2014). However, the concept of “niche-induced oncogenesis” posits that the expression of pro-inflammatory factors by the bone marrow stroma may promote non cell-autonomous malignant transformation in HSPCs. Support for this concept is provided by the leukaemogenic effects of introducing activating Ptpn11 mutations into the stem cell microenvironment in mice (Dong et al., 2016). Germline activating mutations of the protein tyrosine phosphatase SHP2 (encoded by PTPN11) positively regulate Ras signalling in 50% of Noonan syndrome patients, who have an increased risk of developing juvenile myelomonocytic leukaemia (JMML), a childhood myeloproliferative neoplasm (MPN). Remarkably, the introduction of Ptpn11 mutations into murine mesenchymal stem/progenitor cells (MPCs) and osteoprogenitors induced JMML-like MPN and donor-cell-derived MPN following stem cell transplantation as a consequence of excessive production of the pro-inflammatory chemokine CCL3 and the recruitment of monocytes that hyperactivate HSPCs by secreting interleukin 1β.

Is such a mechanism relevant to SDS pathogenesis? Homozygous deletion of the murine *Sbds* gene from *osterix*-expressing mesenchymal stem and progenitor cells (MPCs) induced mitochondrial dysfunction, oxidative stress, increased generation of reactive oxygen species and activated the DNA damage response in HSPCs downstream of p53-S100A8/9-Toll like receptor inflammatory signalling (Zambetti et al., 2016). However, no significant effects on bone marrow cellularity, function or HSPC number were identified, and leukaemia did not develop over the limited 4-week lifespan of the animals. Thus, further experiments are required to further validate the hypothesis that microenvironmental inflammation contributes to clonal selection and transformation in SDS. In particular, it will be important to analyse animals carrying disease-relevant hypomorphic *Sbds* mutations.

HSPCs with impaired translation are likely under strong selective pressure for mutations that suppress the underlying growth defect and reverse the restraints on proliferation. The acquisition of sporadic biallelic *TP53* mutations in SDS is clearly important clinically and biologically for clonal progression and transformation to MDS and is a poor prognostic indicator (Lindsley et al., 2017). Interestingly, the recurrent del(20)q abnormality observed in the bone marrow of SDS patients is commonly associated with loss of the *EIF6* gene (Pressato et al., 2012, Valli et al., 2013). It is attractive to speculate that a reduced dose of eIF6 in SBDS-deficient HSPCs may help bypass the restraint on growth by ameliorating the defect in ribsosomal subunit joining, in turn favouring the formation of actively translating 80S ribosomes. It remains unclear whether the somatic acquisition of *EIF6* gene deletions in SDS has prognostic significance.

Heterozygous somatic mutations in the spliceosome gene *U2AF1* occur in 11% of patients with MDS. Given the links between perturbed ribosome function and MDS (Barlow et al., 2010, Ebert et al., 2008, Finch et al., 2011, Pellagatti and Boultwood, 2017, Shirai et al., 2015), it is intriguing that one consequence of transgenic expression of mutant U2AF1 in the mouse is dysregulation of genes involved in ribosome function and translation. These data suggest that impaired ribosome biogenesis and translation may functionally contribute to mutant U2AF1-associated disease, although this hypothesis needs to be further tested experimentally.

## 16 Diverse functions of genes mutated in human ribosomopathies

The human ribosomopathies are associated with mutations in multiple ribosomal proteins and assembly factors that function at distinct steps in the assembly pathway, evoking a wide range of clinical phenotypes that include haematological malignancies and cancer (Table 1). Acquired hemizygous mutations in the ribosomal protein gene *RPS14* are associated with the 5q-syndrome (Boultwood et al., 2007, Ebert et al., 2008), a subtype of MDS, while pathological variants in *RPL10*, *RPL5* and *RPL22* are found in 10% of cases of paediatric T-ALL (De Keersmaecker et al., 2013, Rao et al., 2012). Acquired somatic mutations in *RPS15* are found in 20% of aggressive relapsed chronic lymphocytic leukaemia, with one third of cases carrying concurrent *TP53* aberrations (Ljungstrom et al., 2016). Among the congenital ribosomopathies, Diamond-Blackfan anaemia (DBA; OMIM #105650, https://www.ncbi.nlm.nih.gov/books/NBK7047/) is a rare, clinically and genetically heterogeneous autosomal dominant disorder that classically presents within the first year of life with normochromic, macrocytic anaemia, reticulocytopenia and reduced or absent erythroid precursors in the bone marrow. Around 50% of individuals have associated short stature and physical anomalies including craniofacial, thumb, cardiac and urogenital defects (Vlachos et al., 2014). DBA is associated with significant predisposition to cancer, with a 30-fold increased risk of developing AML, osteosarcoma or colon cancer (Vlachos et al., 2012). More generally, hemizygous ribosomal protein gene deletions appear to be a common vulnerability in human cancer, especially in conjunction with *TP53* mutations (Ajore et al., 2017). Interestingly, zebrafish hemizygous for loss-of-function mutations in multiple ribosomal proteins develop peripheral nerve sheath tumours (MacInnes et al., 2008), while deletion of *RPS26* induces haematopoietic tumours in Drosophila (Watson et al., 1992), raising the possibility that ribosomal protein genes are evolutionarily conserved tumour suppressors.

**Table 1:** Functions of human ribosomopathy genes.

Disease	Gene	Function in ribosome assembly	Clinical phenotype	References
**Autosomal recessive**				
Shwachman-Diamond syndrome	*SBDS* (yeast *SDO1*)	Cofactor for EFL1 in eIF6 release	Neutropenia, exocrine pancreatic insufficiency, metaphyseal chondrodysplasia, predisposition to MDS/AML	(Finch et al., 2011, Menne et al., 2007, Wong et al., 2011)
	*DNAJC21* (yeast *JJJ1*)	J-protein, HSP70 co-chaperone, Arx1 release		(Meyer et al., 2010)
Bowen-Conradi syndrome	*EMG1* (yeast Nep1)	SPOUT family pseudouridine methyltransferase; required for maturation of 18S rRNA and 40S ribosomal subunit production independent of methyltransferase activity	Growth retardation, psychomotor delay microcephaly, micrognathia, joint contractures, rockerbottom feet	(Meyer et al., 2011, Schilling et al., 2012)
North American Indian Childhood Cirrhosis	*CIRHIN* (yeast *UTP14*)	t-Utp subcomplex of the U3-containing 90S particle; rDNA transcription and 18S pre-rRNA processing	Cirrhosis	(Prieto and McStay, 2007)
Cartilage Hair Hypoplasia	*RMRP*	RNA component of RNase MRP; endonucleolytic cleavage that separates the 18S rRNA from the 5.8S–28S portion of the rRNA precursor	Short limb dwarfism, metaphyseal dysplasia, hypoplastic anaemia, defective B and T cell-mediated immunity, variable intestinal aganglionosis	(Goldfarb and Cech, 2017)
Dyskeratosis congenita, Høyeraal-Hreidarsson syndrome	*PARN*	18S-E pre-rRNA processing	Bone marrow failure, hypomyelination, mucocutaneous defects, pulmonary fibrosis, developmental delay, cerebellar hypoplasia, eosophageal, urethral stenosis	(Ishikawa et al., 2017, Montellese et al., 2017)
Alopecia, neurological and endocrinopathy syndrome	*RBM28* (yeast *NOP4*)	Nucleolar protein, component of 66S pre-ribosomes, 27S pre-rRNA processing	Hair loss, microcephaly, mental retardation, progressive motor retardation, adrenal insufficiency	(Nousbeck et al., 2008, Sun and Woolford, 1994)
**Autosomal Dominant**				
Diamond-Blackfan anaemia	*RPS7, RPS10,* *RPS17, RPS19,* *RPS20, RPS24,* *RPS26, RPS27,* *RPS28, RPS29*	40S subunit proteins	Macrocytic anaemia, craniofacial and thumb abnormalities, short stature, cancer predisposition	(Cmejla et al., 2007, Doherty et al., 2010, Farrar et al., 2008, Gazda et al., 2006, Gazda et al., 2012, Gazda et al., 2008, Gripp et al., 2014, Landowski et al., 2013, Mirabello et al., 2014, Wang et al., 2015)
	*RPL5, RPL11,* *RPL15, RPL19, RPL26, RPL27, RPL31, RPL35A*	60S subunit proteins		
	*GATA1* *TSR2*	Transcription factor eS26 escortin		(Sankaran et al., 2012) (Gripp et al., 2014)
Treacher-Collins syndrome	*TCOF1* *POLR1C* *POLR1D*	rDNA transcription, NBS1 recruitment during DNA damage response	Craniofacial defects, mental retardation	(Ciccia et al., 2014, Larsen et al., 2014)
Congenital asplenia	*RPSA*	40S subunit protein	Absence of spleen	(Bolze et al., 2013)
Aplasia cutis congenita	*BMS1*	Component of the 90S particle, GTPase	Skin agenesis on scalp vertex	(Marneros, 2013)
Familial colorectal cancer type X	*RPS20*	40S subunit protein	Hereditary nonpolyposis colorectal carcinoma with no mismatch repair defects	(Nieminen et al., 2014)
*RPS23*-related ribosomopathy	*RPS23*	40S subunit protein	Microcephaly, hearing loss, intellectual disability, autism	(Paolini et al., 2017)
Leukoencephalopathy, intracranial calcifications and cysts (LCC)	*SNORD118*	box C/D snoRNA U8	Progressive cerebral degeneration	(Jenkinson et al., 2016)
**X-linked recessive**				
Dyskeratosis congenita, Høyeraal-Hreidarsson syndrome	*DKC1*	H/ACA ribonucleoprotein complex subunit 4; rRNA pseudouridine synthase, telomere maintenance	Abnormal skin pigmentation, nail dystrophy, oral leukoplakia, bone marrow failure, cancer predisposition, short stature, microcephaly, immunodeficiency	(Heiss et al., 1998, Lafontaine et al., 1998)
Autism	*RPL10*	60S subunit protein	Microcephaly, growth retardation, seizures	(Brooks et al., 2014)
Microcephaly	*RPL10*		Autism	(Klauck et al., 2006)
**Sporadic**				
Relapsed CLL	*RPS15, RPSA,* *RPS20*	40S subunit proteins, binds MDM2	Adverse prognosis CLL after first line therapy	(Ljungstrom et al., 2015)
Paediatric T-ALL	*RPL5, RPL10,* *RPL22*	60S subunit protein	T-ALL	(De Keersmaecker et al., 2013)
5q- syndrome	*RPS14*	40S subunit protein	MDS and macrocytic anaemia	(Ebert et al., 2008)

The link between DBA and the ribosome was initially established though the identification of pathological variants in the *RPS19* gene that encodes the ribosomal protein RPS19 (Draptchinskaia et al., 1999). Mutations in *RPS19* are found in approximately 25% of DBA cases, but additional autosomal dominant mutations are found in multiple ribosomal protein constituents of both the large and small ribosomal subunits (Table 1). DBA-associated mutations extend beyond ribosomal protein genes to include pathological variants of the X-linked transcription factor GATA1 (Sankaran et al., 2012) and the escortin TSR2 (Gripp et al., 2014) that transfers eS26 (also mutated in DBA) to its binding site on the 90S pre-ribosome in the nucleolus (Schutz et al., 2014). Stabilisation of p53 accounts for a wide range of phenotypes in mice (including defective erythropoiesis, epidermal melanocytosis, reduced body size, disruption of somitogenesis and retinal cell fate determination) associated with a reduced dosage of multiple ribosomal protein genes (Barlow et al., 2010, Jaako et al., 2015, McGowan et al., 2008, Oliver et al., 2004).

The *TCOF1* gene that is predominantly mutated in Treacher-Collins syndrome (TCS, OMIM: 154500) (Bowman et al., 2012) encodes a nucleolar phosphoprotein that has recently been proposed to function as a DNA damage response factor that regulates rDNA transcription by recruiting the Nijmegen breakage syndrome protein NBS1 to nucleoli following DNA damage (Ciccia et al., 2014, Larsen et al., 2014). The discovery of mutations in *POLR1C* and *POLR1D* (Dauwerse et al., 2011) in TCS supports the possibility that defective DNA repair may contribute to disease pathophysiology.

Mutations in *EMG1* (yeast *NEP1*) cause autosomal recessive Bowen-Conradi syndrome (OMIM: 211180) (Armistead et al., 2009). *NEP1* encodes a dimeric N1-specific pseudouridine methyltransferase that is structurally homologous to the SPOUT (S-adenosylmethyltransferase-binding knotted methyltransferase fold, exemplified by SpoU and TrmD) family of methyltransferases (Wurm et al., 2010). However, the key role of Nep1 appears to be related to Rps19 loading, as the methyltransferase activity is not essential for ribosome assembly (Meyer et al., 2011, Schilling et al., 2012).

Homozygous R565W mutations in human UTP4/CIRHIN cause the autosomal recessive disorder North American Indian Childhood Cirrhosis (OMIM: 604901) that is found exclusively in Ojibway-Cree children from a First Nations Canadian population (Chagnon et al., 2002). In yeast, Utp4 was first identified as a component of the SSU processome (Dragon et al., 2002). Utp4 is also part of the t-Utp/UtpA subcomplex of the SSU processome required for pre-rRNA processing and transcription (Gallagher et al., 2004). The UTP4 component of the human t-UTP/UTPA subcomplex is only required for 18S pre-rRNA processing and not pre-rDNA transcription (Prieto and McStay, 2007).

The *PARN* gene, defective in dyskeratosis congenita, Høyeraal-Hreidarsson syndrome and pulmonary fibrosis (Burris et al., 2016, Dhanraj et al., 2015, Stuart et al., 2015, Tummala et al., 2015), encodes a poly(A) specific ribonuclease involved in poly(A) tail shortening of a wide range of mRNA and non-coding RNA substrates including scaRNAs and box H/ACA small nucleolar RNAs (snoRNAs) (Berndt et al., 2012), the human telomerase RNA component (Moon et al., 2015, Nguyen et al., 2015, Tseng et al., 2015), miRNAs (Katoh et al., 2015, Yoda et al., 2013), piRNAs (Izumi et al., 2016, Tang et al., 2016) and Y RNAs (Shukla and Parker, 2017). The functional repertoire for PARN has recently been expanded to include nuclear pre-rRNA processing. PARN is a component of nuclear pre-40S ribosomal particles that is required for 3′-5′ exonucleolytic trimming of the 18S-E pre-rRNA and promotes the subsequent final 3′ end maturation of the 18S rRNA by the endonuclease NOB1 in the cytosol (Ishikawa et al., 2017, Montellese et al., 2017). Thus, PARN, like DKC1, bridges telomere maintenance and ribosome biogenesis in congenital bone marrow failure disorders.

MRP RNA is an abundant, essential, noncoding RNA that is highly conserved in all eukaryotes (Davila Lopez et al., 2009, Piccinelli et al., 2005). Autosomal dominant Cartilage Hair Hypoplasia (CHH, OMIM: 250250), caused by mutations in MRP RNA, is characterised by short stature, sparse hair, malabsorption, haematological and immunological defects and predisposition to cancer (Ridanpaa et al., 2001). In a series of elegant experiments, the elimination of cellular MRP RNA by CRISPR-Cas9 genome editing revealed that MRP catalyses the endonucleolytic cleavage within ITS1 that separates the 18S rRNA from the 5.8S—28S portion of the rRNA precursor (Goldfarb and Cech, 2017).

Box C/D snoRNAs are evolutionarily conserved non-protein-coding RNAs involved in ribosome biogenesis (Watkins and Bohnsack, 2012). Interestingly, biallelic mutations in the *SNORD118* gene that encodes the U8 box C/D snoRNA cause the cerebral microangiopathy known as leukoencephalopathy with calcifications and cysts (LCC) (Jenkinson et al., 2016). LCC is associated with progressive cerebral degeneration that is characterised pathologically by angiomatous-like blood vessels with gliosis and Rosenthal fibre deposition (Labrune et al., 1996). The U8 snoRNA is a vertebrate-specific ribosome assembly factor that is essential for maturation of the 5.8S and 28S rRNAs (Peculis, 1997, Peculis and Steitz, 1994, Tyc and Steitz, 1989). U8 is transcribed independently to generate precursor U8 snoRNAs that are processed to mature box C/D U8 snoRNAs (Peculis et al., 2001). The conserved box C/D motif of U8 recruits four core proteins, 15.5K, NOP56, NOP58 and fibrillarin, that bind in a stepwise manner (Kiss et al., 2006). A conserved LSm (like-Sm) binding site on the U8 snoRNA binds seven individual LSm proteins, forming a ring-like small nucleolar ribonucleoprotein complex that is necessary for ribosomal RNA processing (Kufel et al., 2003). In summary, LCC likely represents a novel form of ribosomopathy caused by the expression of hypomorphic variants of the U8 box C/D snoRNA.

## 17 Future studies

For SDS patients and their families, it will be important to identify informative prognostic biomarkers to predict who is at highest risk of progressing to MDS/AML and who may benefit from early therapeutic intervention. International registries will help expand the range of clinical phenotypes in SDS and define the natural history of the condition. Exome sequencing will likely reveal further gene variants among the 5–10% of individuals clinically diagnosed as SDS who are negative for *SBDS* and *DNAJC21* mutations, while prospective sequencing of serial samples from SDS patients will be important to identify the key genetic changes that promote clonal progression to MDS and leukaemia. The generation of viable SDS animal models will be important both for elucidating mechanisms of disease and for testing novel therapeutics, but significant progress may also come from cellular and induced pluripotent stem cell disease models (Pellagatti et al., 2016). The development of targeted therapeutics will ultimately depend on better understanding of the fundamental molecular mechanisms of cytoplasmic ribosome maturation that are corrupted in SDS. In particular, the recent advances in single-particle cryo-EM are set to rapidly transform our understanding of ribosome assembly and have the potential to provide structural frameworks for drug design. There are a number of outstanding unanswered questions. What is the precise mechanism of eIF6 release and the role of eIF6 phosphorylation? What is the molecular basis of the allosteric modulation of EFL1 function by SBDS and what is the timing and precise role of EFL1 GTP hydrolysis? What is the molecular mechanism of p53 activation in response to eIF6 retention, defective subunit joining and attenuated translation in SBDS-deficient cells? What are the molecular mechanisms that evoke tissue-specific phenotypes downstream of p53? Understanding the molecular pathophysiology of SDS and related human ribosomopathies is an exciting area of research that is likely to provide significant new insights into the fundamental conserved mechanisms of ribosome assembly, its quality control and cancer biology more generally.

## Conflict of interest

None.
